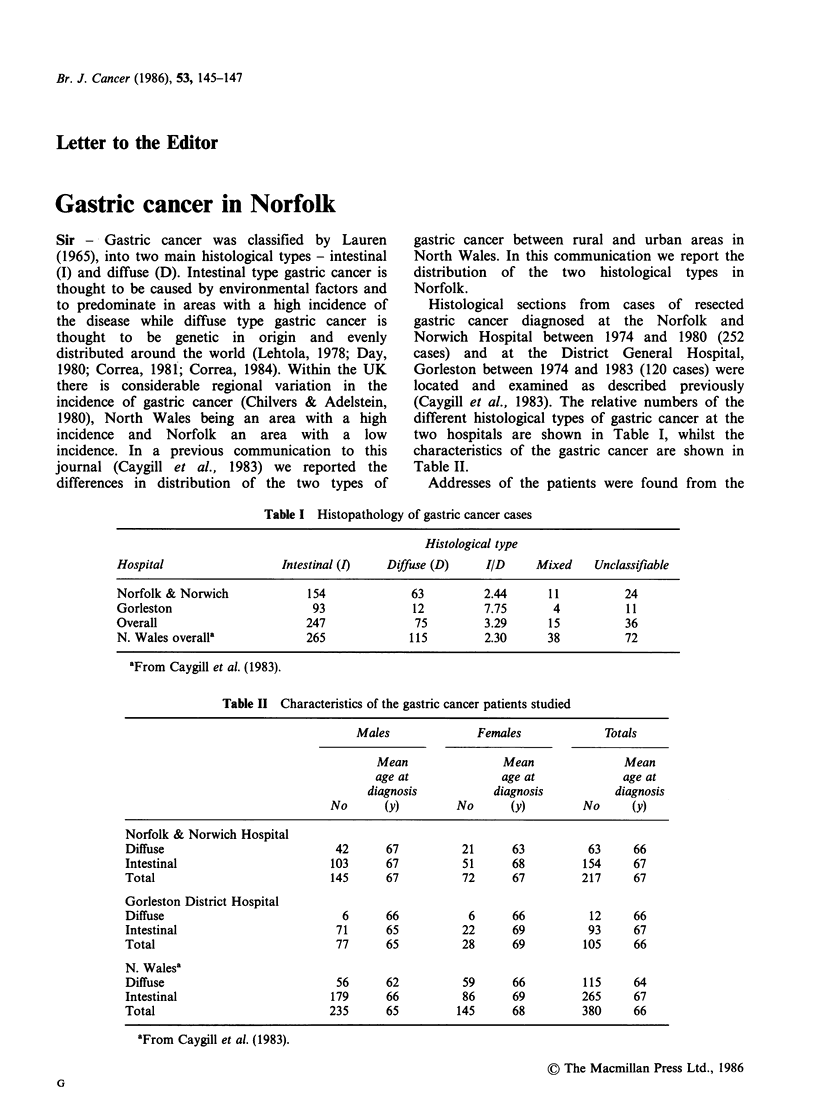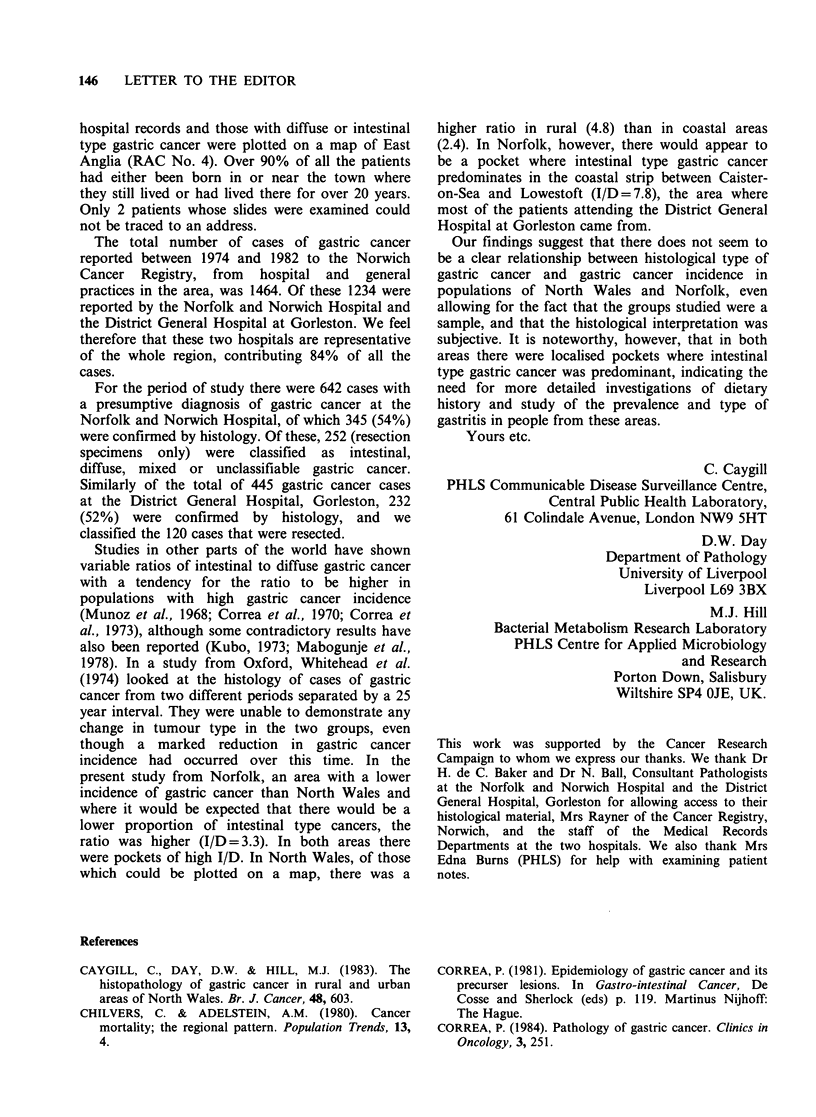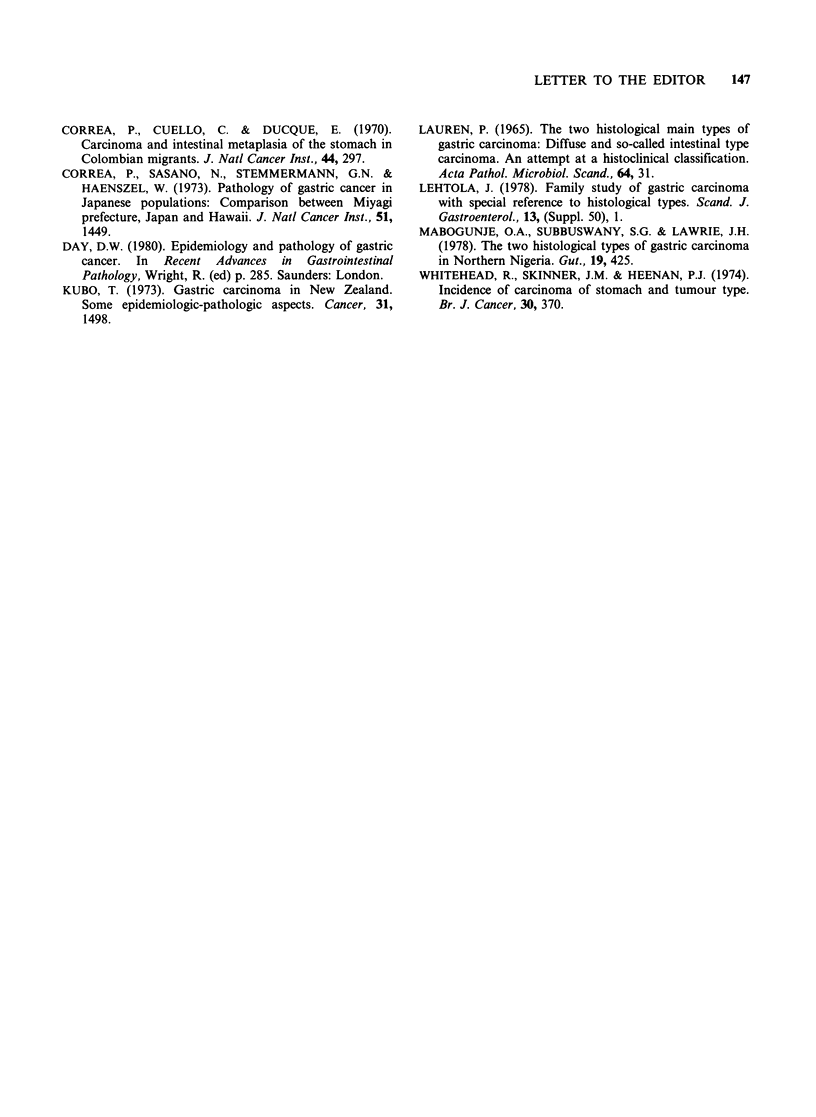# Gastric cancer in Norfolk.

**DOI:** 10.1038/bjc.1986.21

**Published:** 1986-01

**Authors:** C. Caygill, D. W. Day, M. J. Hill


*Br. J. Cancer* (1986), **53**, 145–147

### Letter to the Editor

### Gastric cancer in Norfolk

**Sir** – Gastric cancer was classified by Lauren (1965), into two main histological types – intestinal (I) and diffuse (D). Intestinal type gastric cancer is thought to be caused by environmental factors and to predominate in areas with a high incidence of the disease while diffuse type gastric cancer is thought to be genetic in origin and evenly distributed around the world (Lehtola, 1978; Day, 1980; Correa, 1981; Correa, 1984). Within the UK there is considerable regional variation in the incidence of gastric cancer (Chilvers & Adelstein, 1980), North Wales being an area with a high incidence and Norfolk an area with a low incidence. In a previous communication to this journal (Caygill *et al.,* 1983) we reported the differences in distribution of the two types of gastric cancer between rural and urban areas in North Wales. In this communication we report the distribution of the two histological types in Norfolk.

Histological sections from cases of resected gastric cancer diagnosed at the Norfolk and Norwich Hospital between 1974 and 1980 (252 cases) and at the District General Hospital, Gorleston between 1974 and 1983 (120 cases) were located and examined as described previously (Caygill *et al.,* 1983). The relative numbers of the different histological types of gastric cancer at the two hospitals are shown in Table I, whilst the characteristics of the gastric cancer are shown in Table II.

Addresses of the patients were found from the

**Table I** Histopathology of gastric cancer cases

| | Histological type | | | | |
|---|---|---|---|---|---|
| *Hospital* | *Intestinal (I)* | *Diffuse (D)* | *I/D* | *Mixed* | *Unclassifiable* |
| Norfolk & Norwich | 154 | 63 | 2.44 | 11 | 24 |
| Gorleston | 93 | 12 | 7.75 | 4 | 11 |
| Overall | 247 | 75 | 3.29 | 15 | 36 |
| N. Wales overall[a] | 265 | 115 | 2.30 | 38 | 72 |

[a]From Caygill *et al.* (1983).

**Table II** Characteristics of the gastric cancer patients studied

| | *Males* | | *Females* | | *Totals* | |
|---|---|---|---|---|---|---|
| | *No* | *Mean age at diagnosis (y)* | *No* | *Mean age at diagnosis (y)* | *No* | *Mean age at diagnosis (y)* |
| Norfolk & Norwich Hospital | | | | | | |
| Diffuse | 42 | 67 | 21 | 63 | 63 | 66 |
| Intestinal | 103 | 67 | 51 | 68 | 154 | 67 |
| Total | 145 | 67 | 72 | 67 | 217 | 67 |
| Gorleston District Hospital | | | | | | |
| Diffuse | 6 | 66 | 6 | 66 | 12 | 66 |
| Intestinal | 71 | 65 | 22 | 69 | 93 | 67 |
| Total | 77 | 65 | 28 | 69 | 105 | 66 |
| N. Wales[a] | | | | | | |
| Diffuse | 56 | 62 | 59 | 66 | 115 | 64 |
| Intestinal | 179 | 66 | 86 | 69 | 265 | 67 |
| Total | 235 | 65 | 145 | 68 | 380 | 66 |

[a]From Caygill *et al.* (1983).

© The Macmillan Press Ltd., 1986

hospital records and those with diffuse or intestinal type gastric cancer were plotted on a map of East Anglia (RAC No. 4). Over 90% of all the patients had either been born in or near the town where they still lived or had lived there for over 20 years. Only 2 patients whose slides were examined could not be traced to an address.

The total number of cases of gastric cancer reported between 1974 and 1982 to the Norwich Cancer Registry, from hospital and general practices in the area, was 1464. Of these 1234 were reported by the Norfolk and Norwich Hospital and the District General Hospital at Gorleston. We feel therefore that these two hospitals are representative of the whole region, contributing 84% of all the cases.

For the period of study there were 642 cases with a presumptive diagnosis of gastric cancer at the Norfolk and Norwich Hospital, of which 345 (54%) were confirmed by histology. Of these, 252 (resection specimens only) were classified as intestinal, diffuse, mixed or unclassifiable gastric cancer. Similarly of the total of 445 gastric cancer cases at the District General Hospital, Gorleston, 232 (52%) were confirmed by histology, and we classified the 120 cases that were resected.

Studies in other parts of the world have shown variable ratios of intestinal to diffuse gastric cancer with a tendency for the ratio to be higher in populations with high gastric cancer incidence (Munoz *et al.*, 1968; Correa *et al.*, 1970; Correa *et al.*, 1973), although some contradictory results have also been reported (Kubo, 1973; Mabogunje *et al.*, 1978). In a study from Oxford, Whitehead *et al.* (1974) looked at the histology of cases of gastric cancer from two different periods separated by a 25 year interval. They were unable to demonstrate any change in tumour type in the two groups, even though a marked reduction in gastric cancer incidence had occurred over this time. In the present study from Norfolk, an area with a lower incidence of gastric cancer than North Wales and where it would be expected that there would be a lower proportion of intestinal type cancers, the ratio was higher (I/D = 3.3). In both areas there were pockets of high I/D. In North Wales, of those which could be plotted on a map, there was a higher ratio in rural (4.8) than in coastal areas (2.4). In Norfolk, however, there would appear to be a pocket where intestinal type gastric cancer predominates in the coastal strip between Caister-on-Sea and Lowestoft (I/D = 7.8), the area where most of the patients attending the District General Hospital at Gorleston came from.

Our findings suggest that there does not seem to be a clear relationship between histological type of gastric cancer and gastric cancer incidence in populations of North Wales and Norfolk, even allowing for the fact that the groups studied were a sample, and that the histological interpretation was subjective. It is noteworthy, however, that in both areas there were localised pockets where intestinal type gastric cancer was predominant, indicating the need for more detailed investigations of dietary history and study of the prevalence and type of gastritis in people from these areas.

Yours etc.

C. Caygill
PHLS Communicable Disease Surveillance Centre,
Central Public Health Laboratory,
61 Colindale Avenue, London NW9 5HT

D.W. Day
Department of Pathology
University of Liverpool
Liverpool L69 3BX

M.J. Hill
Bacterial Metabolism Research Laboratory
PHLS Centre for Applied Microbiology
and Research
Porton Down, Salisbury
Wiltshire SP4 0JE, UK.

This work was supported by the Cancer Research Campaign to whom we express our thanks. We thank Dr H. de C. Baker and Dr N. Ball, Consultant Pathologists at the Norfolk and Norwich Hospital and the District General Hospital, Gorleston for allowing access to their histological material, Mrs Rayner of the Cancer Registry, Norwich, and the staff of the Medical Records Departments at the two hospitals. We also thank Mrs Edna Burns (PHLS) for help with examining patient notes.

**References**

CAYGILL, C., DAY, D.W. & HILL, M.J. (1983). The histopathology of gastric cancer in rural and urban areas of North Wales. *Br. J. Cancer*, **48,** 603.

CHILVERS, C. & ADELSTEIN, A.M. (1980). Cancer mortality; the regional pattern. *Population Trends*, **13,** 4.

CORREA, P. (1981). Epidemiology of gastric cancer and its precurser lesions. In *Gastro-intestinal Cancer*, De Cosse and Sherlock (eds) p. 119. Martinus Nijhoff: The Hague.

CORREA, P. (1984). Pathology of gastric cancer. *Clinics in Oncology*, **3,** 251.

CORREA, P., CUELLO, C. & DUCQUE, E. (1970). Carcinoma and intestinal metaplasia of the stomach in Colombian migrants. *J. Natl Cancer Inst.*, **44**, 297.

CORREA, P., SASANO, N., STEMMERMANN, G.N. & HAENSZEL, W. (1973). Pathology of gastric cancer in Japanese populations: Comparison between Miyagi prefecture, Japan and Hawaii. *J. Natl Cancer Inst.*, **51**, 1449.

DAY, D.W. (1980). Epidemiology and pathology of gastric cancer. In *Recent Advances in Gastrointestinal Pathology*, Wright, R. (ed) p. 285. Saunders: London.

KUBO, T. (1973). Gastric carcinoma in New Zealand. Some epidemiologic-pathologic aspects. *Cancer*, **31**, 1498.

LAUREN, P. (1965). The two histological main types of gastric carcinoma: Diffuse and so-called intestinal type carcinoma. An attempt at a histoclinical classification. *Acta Pathol. Microbiol. Scand.*, **64**, 31.

LEHTOLA, J. (1978). Family study of gastric carcinoma with special reference to histological types. *Scand. J. Gastroenterol.*, **13**, (Suppl. 50), 1.

MABOGUNJE, O.A., SUBBUSWANY, S.G. & LAWRIE, J.H. (1978). The two histological types of gastric carcinoma in Northern Nigeria. *Gut.*, **19**, 425.

WHITEHEAD, R., SKINNER, J.M. & HEENAN, P.J. (1974). Incidence of carcinoma of stomach and tumour type. *Br. J. Cancer*, **30**, 370.